# A novel pipeline for prioritizing cancer type‐specific therapeutic vulnerabilities using DepMap identifies PAK2 as a target in head and neck squamous cell carcinomas

**DOI:** 10.1002/1878-0261.13558

**Published:** 2023-12-13

**Authors:** Malay K. Sannigrahi, Austin C. Cao, Pavithra Rajagopalan, Lova Sun, Robert M. Brody, Lovely Raghav, Phyllis A. Gimotty, Devraj Basu

**Affiliations:** ^1^ Department of Otorhinolaryngology‐Head and Neck Surgery University of Pennsylvania Philadelphia PA USA; ^2^ Department of Medicine University of Pennsylvania Philadelphia PA USA; ^3^ Department of Biostatistics, Epidemiology and Informatics University of Pennsylvania Philadelphia PA USA; ^4^ Ellen and Ronald Caplan Cancer Center The Wistar Institute Philadelphia PA USA

**Keywords:** CRISPR, DepMap, head and neck squamous cell carcinoma, PAK2, targeted therapy, wild type p53

## Abstract

There is limited guidance on exploiting the genome‐wide loss‐of‐function CRISPR screens in cancer Dependency Map (DepMap) to identify new targets for individual cancer types. This study integrated multiple tools to filter these data in order to seek new therapeutic targets specific to head and neck squamous cell carcinoma (HNSCC). The resulting pipeline prioritized 143 targetable dependencies that represented both well‐studied targets and emerging target classes like mitochondrial carriers and RNA‐binding proteins. In total, 14 targets had clinical inhibitors used for other cancers or nonmalignant diseases that hold near‐term potential to repurpose for HNSCC therapy. Comparing inhibitor response data that were publicly available for 13 prioritized targets between the cell lines with high vs. low dependency on each target uncovered novel therapeutic potential for the PAK2 serine/threonine kinase. *PAK2* gene dependency was found to be associated with wild‐type p53, low *PAK2* mRNA, and diploid status of the 3q amplicon containing *PAK2*. These findings establish a generalizable pipeline to prioritize clinically relevant targets for individual cancer types using DepMap. Its application to HNSCC highlights novel relevance for PAK2 inhibition and identifies biomarkers of PAK2 inhibitor response.

## Graphical abstract

This study provides a template for using the public CRISPR screen data in DepMap to identify novel therapeutic targets for specific cancer types and rapidly advance them toward clinical application. As an example, we used the gene dependency data in DepMap for HPV‐negative head and neck cancer to demonstrate application of the pipeline, which integrates the CRISPR data with multiple other publicly available resources to identify the most promising targets.

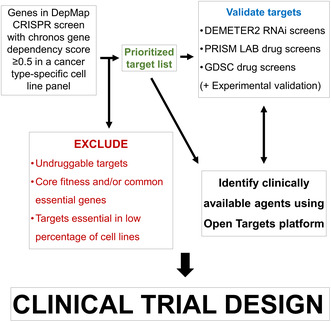

**Abbreviations**
ANOVA: analysis of variance
CRISPR: Clustered Regularly Interspaced Short Palindromic Repeats
DAVID: Database for Annotation, Visualization, and Integrated Discovery
DGIdb: Drug Gene Interaction Database
ESCC: oesophageal squamous cell carcinoma
GDSC: Genomics of Drug Sensitivity in Cancer
GISTIC: Genomic Identification of Significant Targets in Cancer
HNSCC: head and neck squamous cell carcinoma
HPV: human papilloma virus
IQR: interquartile range
n.s.: not significant
PAK2: P21 Activated Kinase 2
PRISM: Profiling Relative Inhibition Simultaneously in Mixtures
RSEM: RNA‐Seq by Expectation–Maximization
TPM: transcripts per million

## 1 Introduction

Results from CRISPR/Cas9 genome‐wide loss‐of‐function screens are now publicly available for 1865 cancer cell lines on the Cancer Dependency Map (DepMap) portal [1], which also provides analytic and visualization tools for this expansive dataset. DepMap reports the effect of deleting each gene on cell line viability using the Chronos gene effect score [2], which normalizes effect sizes using the distributions of non‐essential and pan‐essential genes and designates stronger gene dependencies with lower scores. Chronos gene effect scores derive from a mathematical model that integrates screen results from multiple biologic replicates while accounting for variations in cell population dynamics over time upon deleting different genes and addressing other sources of bias and noise. Gene probability score, a second metric provided by DepMap, measures how likely a given gene effect score is to be part of the essential distribution by accounting variations in screen quality among cell lines. The DepMap Portal also incorporates tools to analyse relationships between identified gene dependencies and their mRNA expression, copy number variations, somatic mutations, and gene fusions in the cell lines. The portal also facilitates further evaluation of gene dependencies identified by CRISPR by facilitating comparison to prior RNAi screens.

DepMap has offered new insight into the landscape of gene dependencies across human cancers [3] and detected potential synthetic lethalities linked to specific somatic mutations [4], copy number alterations [5], and gene fusions [6]. However, the published analyses of DepMap CRISPR screen data have mostly been conducted on a pan‐cancer basis and offer limited guidance for pursuing therapeutic vulnerabilities and predictive biomarkers specific to individual cancer types. Furthermore, analyses of individual cancer types in DepMap to date have narrowly focused on features distinguishing a malignancy of interest from the pan cancer dataset [7, 8, 9], predetermined biological processes [10, 11, 12, 13], or developing prognostic models [14, 15, 16, 17].

The potential utility of DepMap in therapeutic innovation for individual cancer types is explored in a novel manner in this study using head and neck squamous cell carcinomas (HNSCCs), the 6th most common cancer globally [18]. The human papilloma virus negative (HPV(−)) subtype of HNSCC remains the most common form of this disease [18] and is well‐represented by cell line models in DepMap. For decades, patients with advanced HPV(−) HNSCCs have been standardly treated with combinations of surgery, external beam radiation, and cytotoxic chemotherapy. Despite receiving aggressive multimodality therapy, these patients continue to suffer high rates of cancer recurrence and mortality along with lifelong disabilities created by treatment toxicity [19]. Few modern targeted therapies for HPV(−) HNSCC have emerged, and the approved therapies targeting EGFR [20] and the PD1/PDL1 axis [21] have had limited overall impact on treatment outcomes. The DepMap CRISPR screen data specific to HPV(−) HNSCC remains to be analysed systematically for potential therapeutic vulnerabilities and genetic biomarkers of therapy response that may lead to more effective and less toxic treatments.

This study sought to integrate the gene dependency data for HPV(−) HNSCC in DepMap with multiple other public resources to create a list of targets for this disease with highest priority for further development. For each gene dependency, our prioritization pipeline considered druggability of the target, current status of drug development against it, existing experimental inhibitor response data, potential toxicity to normal cells, and genetic traits associated with favourable responses. This information is cataloged by us in a form suitable for guiding efforts toward drug discovery, preclinical therapeutic testing, and clinical trial design. Our findings identify several targets with drugs that have been used clinically for other diseases and thus could readily be repurposed for HPV(−) HNSCC. We also describe novel utility for targeting the PAK2 serine–threonine kinase, which presently lacks a clinical inhibitor, along with potential biomarkers of favourable anti‐PAK2 therapy responses. In doing so, our results establish a rational approach to interpreting and filtering the DepMap CRISPR screen data for HPV(−) HNSCC that is also generalizable other specific cancer types and ongoing future updates of the multiple datasets used here.

## 2 Materials and methods

### 2.1 Cell line data analysis

Gene dependency data processed by the Chronos algorithm was extracted initially from the 22Q1 public data release from the DepMap at the Broad Institute [1, 2] for all HPV(−) HNSCC (*n* = 63) and oesophageal squamous cell carcinoma (ESCC) (*n* = 24) cell lines in this resource. A re‐analysis prior to publication was performed with the 23Q2 release. The Drug Gene Interaction Database (DGIdb) [22] was used to filter for the genes predicted to encode for druggable proteins. The DAVID Gene Functional Classification Tool [23] was used to classify targetable dependencies based on function. The Open Targets Platform [24] was used to identify approved or investigational drugs known to target druggable proteins and describe their application in clinical trials to date along with their FDA approval status. Mutation and copy number data from whole exome sequencing along with RNAseq‐based gene expression data (TPM + 1) were extracted from the cancer cell line encyclopedia (CCLE) [3] via the DepMap data portal. Drug response information was sourced from the GDSC2 dataset of the Genomics of Drug Sensitivity in Cancer database (GDSC, Release 8.3) [25]. Additional drug response information and RNAi screen results were obtained from DepMap PRISM Repurposing Public 23Q2 dataset and Gene Effect RNAi (DEMETER2) data, respectively, via the DepMap data portal. The R code used to prioritize dependencies and visualize them as a dot plot showing the number of cell lines with each dependency vs. median gene effect score in those cell lines is available in the Github repository at https://github.com/BasuLab2023/DepMap-2023.

### 2.2 TCGA data analysis

Data for the Head and Neck Squamous Cell Carcinoma TCGA cohort (project TCGA‐HNSCC, *n* = 523 cases) were downloaded from the Genomic Data Commons via cBioPortal [26] and includes mRNA expression (RSEM), putative copy‐number alterations (GISTIC), log_2_ gene copy number, mutations, and overall survival. The 415 HPV‐negative cases were selected from the broader cohort by excluding the 72 tumours that were HPV‐positive and 36 where HPV status was unknown based on mapping of > 1000 RNA sequencing reads aligning to high‐risk HPV E6 and/or E7 [27].

### 2.3 Statistical analyses

Significance was evaluated using 2‐tailed Mann–Whitney *U* test or unpaired Welch's *t*‐test when variances were unequal when comparing 2 groups for gene expression, copy number, or drug response. Pearson correlation coefficients were derived from analysing the means. Relationships between cancer‐related mutations and gene dependencies were defined using two‐sample *t*‐tests that compared the median gene effect score between cell lines with and without the cancer‐related mutation of interest. A relationship was designated as significant if *P* < 0.05 and Cohen's *d* effect size was ≥ 1 or ≤ −1. Log‐rank tests were used to compare group‐specific Kaplan–Meier survival curves. For multiple comparisons of drug responses in cell lines after 1‐way ANOVA, *P* values were computed using Dunnett's or Holm‐Šidák procedure. Tests used are indicated in figure legends. Analyses were performed using prism (graphpad Software). A *P* value less than 0.05 was considered significant.

## 3 Results

### 3.1 Identification and prioritization of targetable dependencies in cell line models of HPV(−) HNSCC

**Fig. 1:**
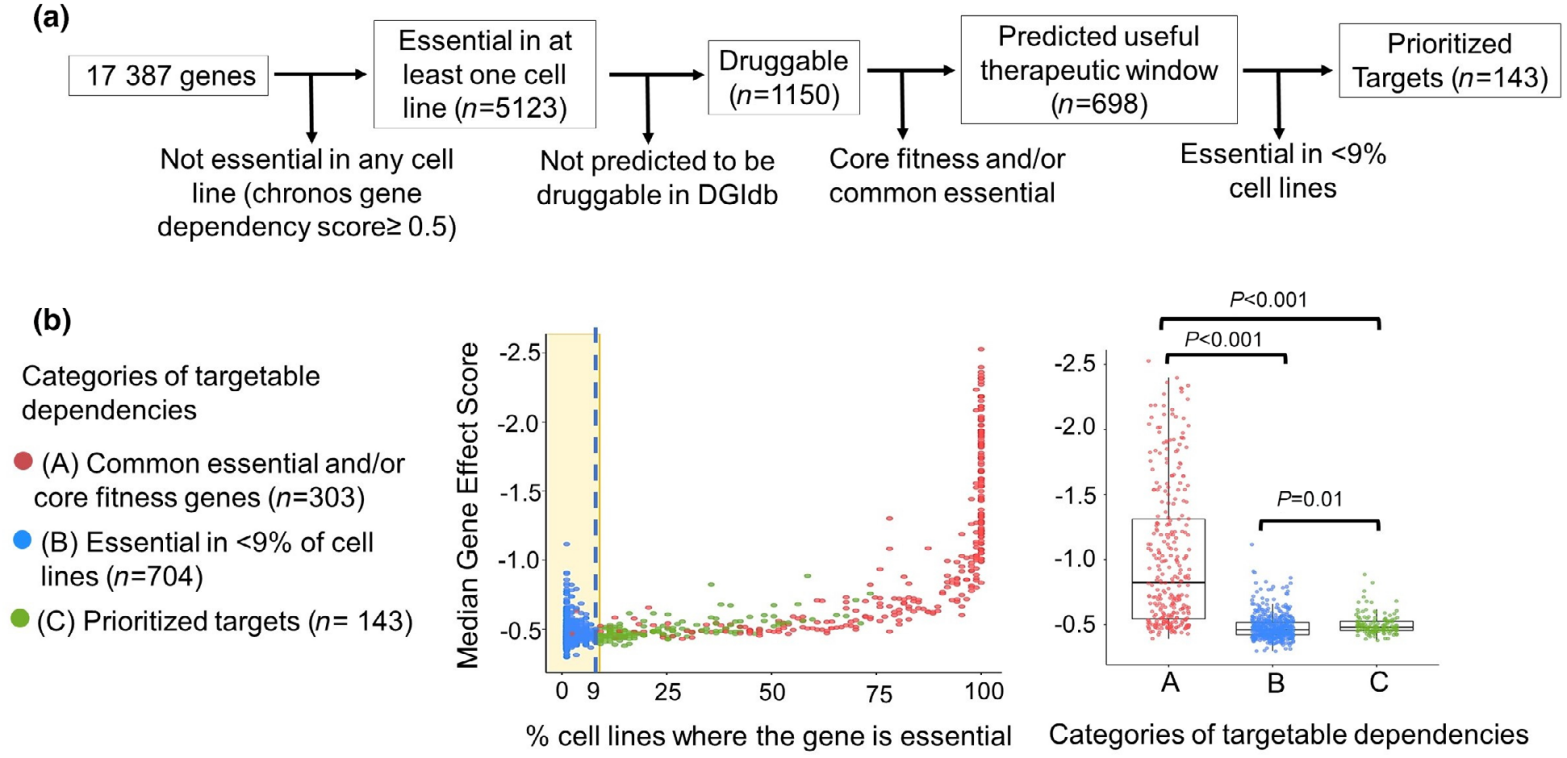
Identification and prioritization of targetable dependencies in HPV(−) HNSCC. (a) Overall pipeline for prioritization of essential genes in HPV(−) HNSCC cell line models in DepMap. (b) Median gene effect of identified druggable dependencies (*n* = 1001) vs. percentage of cell lines where the gene is essential (left) and the distribution of median gene effect scores in prioritized vs. deprioritized categories of targets (right). Genes that are essential in < 9% cell lines are demarcated in the area within the yellow shading and vertical broken line. Adjusted *P* values were defined by one‐way Welch's ANOVA corrected with Dunnett's multiple comparisons test.

The 63 HPV‐negative HNSCC cell lines annotated in DepMap were selected for analysis, and the size of this panel was increased to 87 by adding all 24 cell lines from oesophageal squamous cell carcinomas (ESCCs), which are nearly identical to HPV(−) HNSCCs in their aetiology, tissue of origin, and genetic landscape [28]. The overall filtration algorithm used for the 17 387 genes targeted in the DepMap CRISPR screens is shown in Fig. 1A. Applying the gene probability score cutoff used by DepMap to designate a gene as essential (≥ 0.5) [29] identified 5123 genes that were essential in at least one of the 87 cell lines. The 5123 genes were filtered to the subset of 1001 predicted by the Drug‐Gene Interaction Database to have a protein product that is therapeutically targetable [22]. These 1001 genes are visualized on a scatter plot representing the percentage of cell lines where a gene was essential vs. the median gene effect score across the cell line panel (Fig. 1B, left). To provide further prioritization guided by the scatter plot distribution, we first sought to remove gene dependencies that are shared by normal tissue and thus would not provide a useful therapeutic window. The 1924 dependencies that are present across > 90% of all 990 cancer cell lines in DepMap have been designated as “common essential” genes [29] that are predicted not to be cancer‐specific. 256 common essential genes were present in the list of 1001 targetable dependencies for HNSCC. These genes clustered to the right of the scatter plot with a median gene effect score of −0.95 (IQR: −0.62, −1.51), consistent with the scaling of DepMap gene effect scores to −1.0 as the median for common essential dependencies. To further exclude dependencies shared by normal tissue, we removed genes identified as dependencies across multiple normal cell lineages in prior pooled loss‐of‐function CRISPR screens [30, 31]. Of the 1580 such “core fitness” genes designated by this study, 190 were present in the list of 1001 targetable dependencies. The 190 core fitness genes had a median gene effect score of −0.92 (IQR: −0.61, −1.44), and the overlap of 143 of these genes with the 256 common essential genes provided cross‐validation of two independent approaches for excluding targets likely to have a poor therapeutic window. The two groups together comprised 303 unique genes, which were removed from the 1001 targetable dependencies. Next, we considered the large cluster of genes (*n* = 555) that appeared in < 9% of the cell line panel (Fig. 1B, left) and had significantly lower median effect scores as a group (Fig. 1B, right). This group was also noted to contain outliers with high median effect scores, which were interpreted as most likely arising from stochastic effects due to the small sample size (< 8 cell lines), further supporting exclusion of the group. The remaining 143 genes that were prioritized for further analysis are described in Table S1, where they are ranked by frequency of essentiality and secondarily by median gene effect score.

### 3.2 Functional classification of prioritized targetable dependencies

The 143 prioritized genes were evaluated for recurring functional roles in order to identify broad classes of drug targets meriting preclinical therapeutic evaluation in HNSCC. Application of the Gene Functional Classification Tool of DAVID Bioinformatics Resources [32] with a medium stringency classification (kappa similarity threshold ≥ 0.30) and enrichment score threshold of ≥ 1 highlighted four groups with shared functional annotation (Table 1). The group of receptor tyrosine kinases included IGF1R and multiple ErbB family members, which have already been extensively studied in HNSCC and other solid tumours [33]. The largest group of enriched dependencies consisted of 8 serine/threonine kinases that have diverse signalling functions. This group notably included three Rho/Rac effector proteins: (a) the *MAP3K11* gene product, mixed lineage kinase 3 (MLK3), which is an upstream regulator of MAPK signalling [34] and phosphorylates IκB kinase (IKK) α and β, thereby activating NF‐κB (11), (b) PKN2, a promoter of cell cycle progression previously been explored as a target in HNSCC [35], and (c) PAK2, a multi‐function kinase that integrates cellular stress responses with activation of oncogenic signalling pathways [36] and proved to be of particular interest in subsequent analyses in this study. The other two enriched functional groups were comprised of mitochondrial carrier proteins and RNA‐binding proteins, which have only recently drawn interest as potential therapeutic targets in cancer. The mitochondrial carriers included three members of the SLC25 family, which transport nutrients across the mitochondrial inner membrane, and their role in reprogramming tumour metabolism through overexpression have made them a new focus of preclinical therapeutic development [37]. The functional group of RNA binding proteins included ELAVL1, ZRANB2 and HNRNPA1, whose role in alternative splicing of transcripts to serve tumour development have made them a focus of recent efforts to target altered RNA splicing in cancer [38, 39]. Together, these findings both confirm that our prioritization pipeline captures known targets in HNSCC and underscore the relevance of emerging target classes for other malignancies to this cancer type.

**Table 1:** Functional groups enriched among the 143 prioritized dependencies.

Functional classification	Enrichment score	Genes (Median gene effect score, percentage cell lines)
Serine/threonine kinases	2.57	*MAP3K11* (−0.61, 25%), *PKN2* (−0.58, 36%), *MARK2* (−0.55, 29%), *PAK2* (−0.52, 24%), *CDK8* (−0.46, 13%), *MAP4K2* (−0.45, 15%), *RPS6KA4* (−0.42, 9%), *RIPK3* (−0.5, 25%), *GRK* (−0.48, 22%)
Tyrosine kinases	2.43	*EGFR* (−0.60, 66%), *ERBB2* (−0.59, 45%), *ERBB3* (−0.55, 41%), *IGF1R* (−0.54, 14%), *TYRO3* (−0.49, 23%)
Mitochondrial carriers	1.42	*SLC25A33* (−0.47, 16%), *SLC25A1* (−0.44, 15%), *MTCH2* (−0.41, 9%), *SLC25A25* (−0.39, 9%), *SLC22A25* (−0.44, 11%), *MRGPRX3* (−0.43, 9%), *SPNS1* (−0.54, 28%)
RNA‐binding proteins	1.13	*RBM10* (−0.54, 53%), *PTBP1* (−0.53, 40%), *ELAVL1* (−0.49, 22%), *HNRNPA1* (−0.47, 46%), *ZRANB2* (−0.47, 37%), *RBM5* (−0.47, 11%)

### 3.3 Several prioritized targets have clinical inhibitors that are positioned to repurpose for HNSCC

The subset of the 143 prioritized targets that are best positioned for near‐term testing in HNSCC patients were cataloged by using the Open Targets Database [24] to identify the targets with drugs that have been in clinical trials for other cancers and/or nonmalignant diseases but not HNSCC. Using this tool to filter for inhibitors that have reached at least a phase II trial for any disease identified existing agents for 23 of the 143 targetable proteins. This list contained 9 targets with drugs in active or previous trials for HNSCC (Table S2), including current standard agents like cetuximab, taxanes, and 5‐fluorouracil. Capturing these currently used drugs supported potential clinical utility of the 14 targets with inhibitors have only been studied clinically in other malignant and/or non‐malignant diseases (Table 2) but not HNSCC. These findings support the utility of our prioritization pipeline in identifying clinically relevant targets, including many with inhibitors that have already been used clinically and thus could be repurposed in the near term in HNSCC.

**Table 2:** Prioritized gene products with clinical inhibitors not well‐studied for HNSCC.

Gene symbol	Gene name	Most advanced status of inhibitor	Approved indications	Approved agents	Phase II/III trials for other diseases	Phase II/III agents
*PCSK9*	Proprotein convertase subtilisin/kexin type 9	Approved	Hypercholesterolemia, Cardiovascular disease, metabolic disorders and other non‐malignant diseases	Evolocumab, Alirocumab	Non‐small cell lung cancer (NSCLC), numerous non‐malignant diseases	Alirocumab, Inclisiran
*UGCG*	UDP‐glucose ceramide glucosyltransferase	Approved	Niemann‐Pick disease, Type I Gaucher disease	Miglustat, Eliglustat	Other glycogen storage diseases, cystic fibrosis, HIV	Lucerastat
*IMPDH1*	Inosine monophosphate dehydrogenase 1	Approved	AML, acute nonlymphocytic leukaemias, non‐malignant diseases	Mycophenolate mofetil, Thioguanine, Ribavirin	Other types of leukaemia and lymphoma, non‐malignant diseases	—
*HCRTR1*	Hypocretin receptor 1	Approved	Insomnia	Suvorexant, Lemborexant	Other sleep disorders	Almorexant, Daridorexant, Filorexant
*GANAB*	Glucosidase II alpha subunit	Approved	Type 2 diabetes	Acarbose, miglitol	—	—
*TNFRSF8*	TNF receptor superfamily member 8	Approved	Lymphomas	Brentuximab vedotin	Other types of leukaemia and lymphoma	—
*EGLN1*	egl‐9 family hypoxia inducible factor 1	Approved^a^	Chronic kidney disease, anaemia	Roxadustat	Myelodysplastic syndrome, numerous non‐malignant diseases	Roxadustat Vadadustat, Daprodustat, others
*P2RY6*	Pyrimidinergic receptor P2Y6	Approved^a^	Dry eye syndrome	Diquafosol		
*LTB4R2*	Leukotriene B4 receptor 2	Phase III	—	—	Bullous pemphigoid	Nomacopan
*BIRC2*	Baculoviral IAP repeat containing 2	Phase II	—	—	Ovarian cancer	Birinapant
*ITGB1*	Integrin subunit beta 1	Phase II	—	—	Renal cell carcinoma, pancreatic carcinoma, melanoma, NSCLC	Volociximab, ATN‐161
*MAP3K11*	Mitogen‐activated protein kinase kinase kinase 11	Phase II	—	—	Parkinson disease	CEP‐1347
*LDHA*	Lactate dehydrogenase A	Phase II	—	—	NSCLC, adrenal cortical carcinoma, hyperoxaluria	Nedosiran, AT‐101 (Gossypol)
*PTPN1*	Protein tyrosine phosphatase non‐receptor type 1	Phase II	—	—	Type 2 diabetes	Ertiprotafib, Trodusquemine

^a^ Initial provisional approval revoked.

### 3.4 Drug responses of HNSCC models validate known targets and as well as the novel target PAK2

**Fig. 2:**
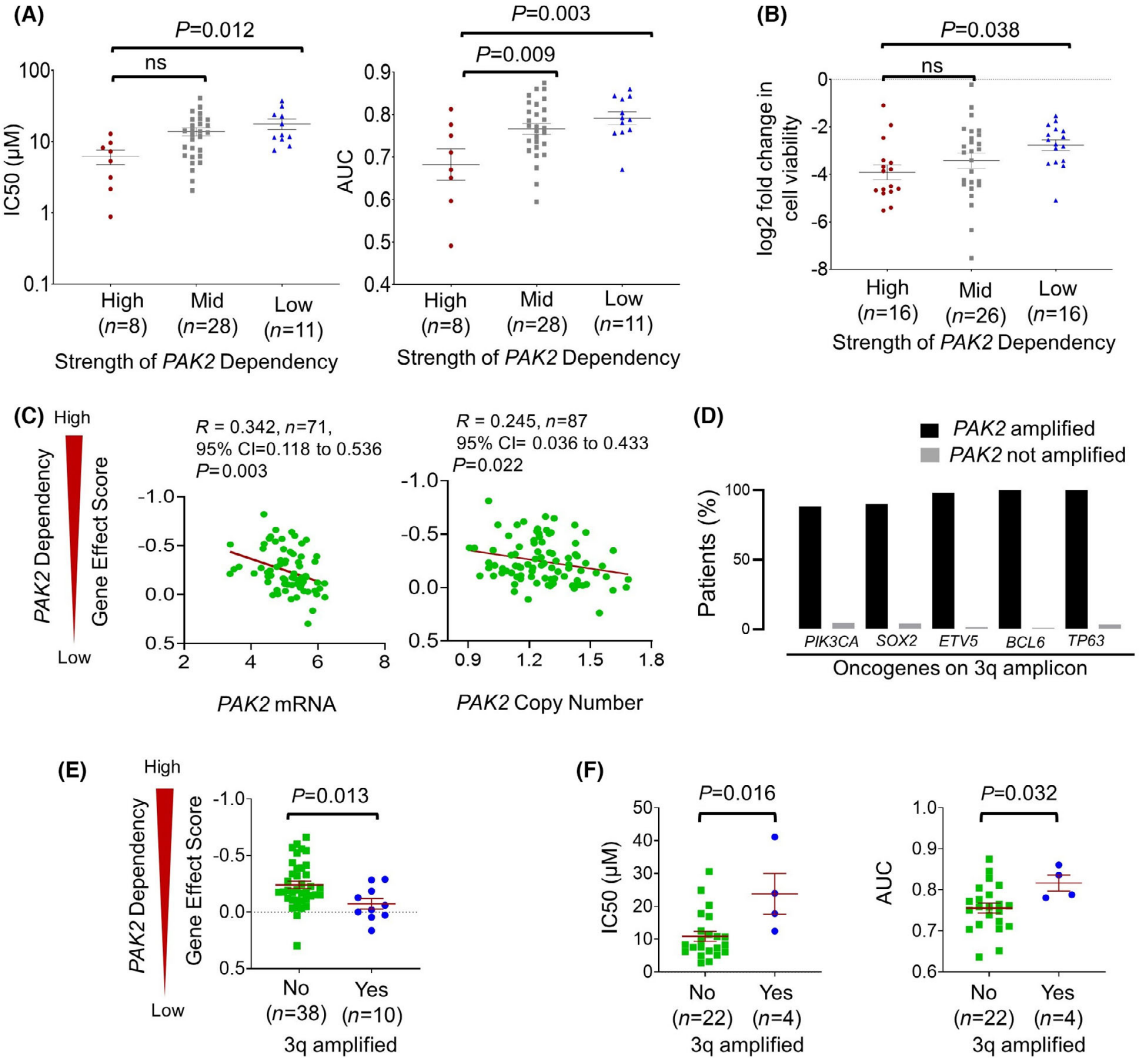
PAK2 inhibitor response vs. PAK2 dependency, genetic alteration, expression, and 3q status. (A) PAK‐5339 and (B) FRAX486 inhibitor responses *in vitro* in GDSC vs. strength of *PAK2* dependency in cell line models of HPV(−) HNSCC. High and low dependencies are defined by the top and bottom quartile of gene effect scores, respectively. Adjusted *P* values were defined by one‐way Welch's ANOVA corrected with Dunnett's multiple comparisons test. (C) *PAK2* dependency vs. mRNA expression and copy number in the cell line models. Pearson correlation coefficients were used to calculate *r* values, and *P* value was determined by *t* distribution. (D) Frequency of co‐amplification of other oncogenes on 3q with *PAK2* in the HPV(−) HNSCCs in TCGA (*n* = 415). (E) *PAK2* dependency in cell lines with ABSOLUTE copy number data (*n* = 48) stratified by presence or absence of amplified 3q. (F) PAK‐5339 responses in subset of cell lines in (E) with inhibitor data in GDSC (*n* = 26). *P* values were calculated by Mann–Whitney *U* test.

The GDSC dataset [25] was used to pursue validation of select prioritized targets. Response profiles for HNSCC cell lines were available in this resource for 39 compounds that inhibit 13 targets in the list of 143 prioritized dependencies. The cell lines were divided into groups with high dependency (top quartile), intermediate dependency (middle half), and low dependency (bottom quartile) for each target based on Chronos Gene Effect Score [2]. The cell lines with high dependency showed significantly stronger *in vitro* growth inhibition when treated with 12 drugs that inhibit one of five targets in the list of 13 targets tested. Four of these five proteins were the well‐studied gene products of *EGFR*, *ERBB2*, *ERBB3*, and *PIK3CA* (Fig. S1). The fifth target to be validated by this method was PAK2 (Fig. 2A), a far less studied serine–threonine kinase that lacks a clinical inhibitor but was inhibited in GDSC data using the PAK‐5339 tool compound. Complete PAK‐5339 dose response curves in GDSC were available for four HNSCC cell line models in the top quartile for *PAK2* dependency and for eight models in the bottom quartile. These 12 dose curves are shown in Fig. S2, which illustrates a clear separation of responses between the groups with high vs. low dependency based on DepMap CRISPR results. An additional PAK2 tool inhibitor (FRAX486) in the PRISM Lab drug screen data [40], which was recently integrated into DepMap, provided further pharmacologic evidence to support this dependency (Fig. 2B). Likewise, additional genetic evidence was provided by older RNAi screen data in DepMap, which showed the HNSCC models found to be *PAK2*‐dependent by CRISPR also to be more susceptible to siRNA silencing of *PAK2* (Fig. S3). The magnitude of *PAK2* gene effect scores in the 24% of cell lines meeting the dependency threshold is contextualized by comparison to those for the well‐studied targets (Fig. S4). Distributions among these dependencies were similar, with only EGFR showing significantly stronger dependency than PAK2. Together, these results support the effectiveness of our prioritization pipeline, which not only captured well‐studied clinical drug targets but also highlighted PAK2 as an emerging target with potential utility for HNSCC therapy.

### 3.5 Low PAK2 mRNA and diploid status of its 3q amplicon predict favourable PAK2 inhibitor responses

Additional data sets were integrated to evaluate whether *PAK2* genetic alterations and/or expression levels are predictors of PAK2 inhibitor responses that might prove clinically useful in HNSCC. Whereas *PAK2* is rarely mutated in cancer, it is frequently amplified and/or overexpressed [41]. *PAK2* copy number and mRNA levels both inversely correlated with *PAK2* gene dependency in the HNSCC cell line models annotated in DepMAP, where cell lines with high dependency had both lower PAK2 expression and diploid gene status (Fig. 2C). Copy gains in *PAK2* were detected in 70% of the 415 HPV(−) HNSCCs in TCGA based on GISTIC score [42], with 12.3% also meeting the GISTIC score threshold for amplification [43]. *PAK2* notably resides on the 3q amplicon containing the oncogenes *PIK3CA*, *SOX2*, *ETV5*, *BCL6*, and *TP63*. These five other genes were consistently co‐amplified with *PAK2* (Fig. 2D), suggesting that absence of 3q amplification predicts higher *PAK2* gene dependency and more favourable inhibitor responses. This possibility was tested using the 48 HPV(−) cell line models of HNSCC whose copy number data is reported in DepMap. Whereas most of these cell lines (*n* = 47, 98%) had 3q copy gains, the subset meeting the threshold for amplification defined by the ABSOLUTE package [44, 45] (*n* = 10, 21%) had significantly reduced dependency on *PAK2* (Fig. 2E). Likewise, among the 26 of 48 cell lines with drug response data in GDSC, the 4 lines (15.4%) with 3q amplification exhibited significantly weaker responses to the small molecule PAK2 inhibitor PAK‐5339 (Fig. 2F). Of note, the HPV(−) HNSCC patients in TCGA with 3q copy gain or amplification showed no differences in overall survival relative to 3q diploid cases (Fig. S5). The findings underscore potential relevance of PAK2 inhibitors for the large fraction of HPV(−) HNSCCs that maintain diploid 3q and carry similarly poor prognosis to those with *PAK2* copy gains via the 3q amplicon.

### 3.6 Wild type p53 predicts favourable PAK2 inhibitor responses in HNSCC

**Fig. 3:**
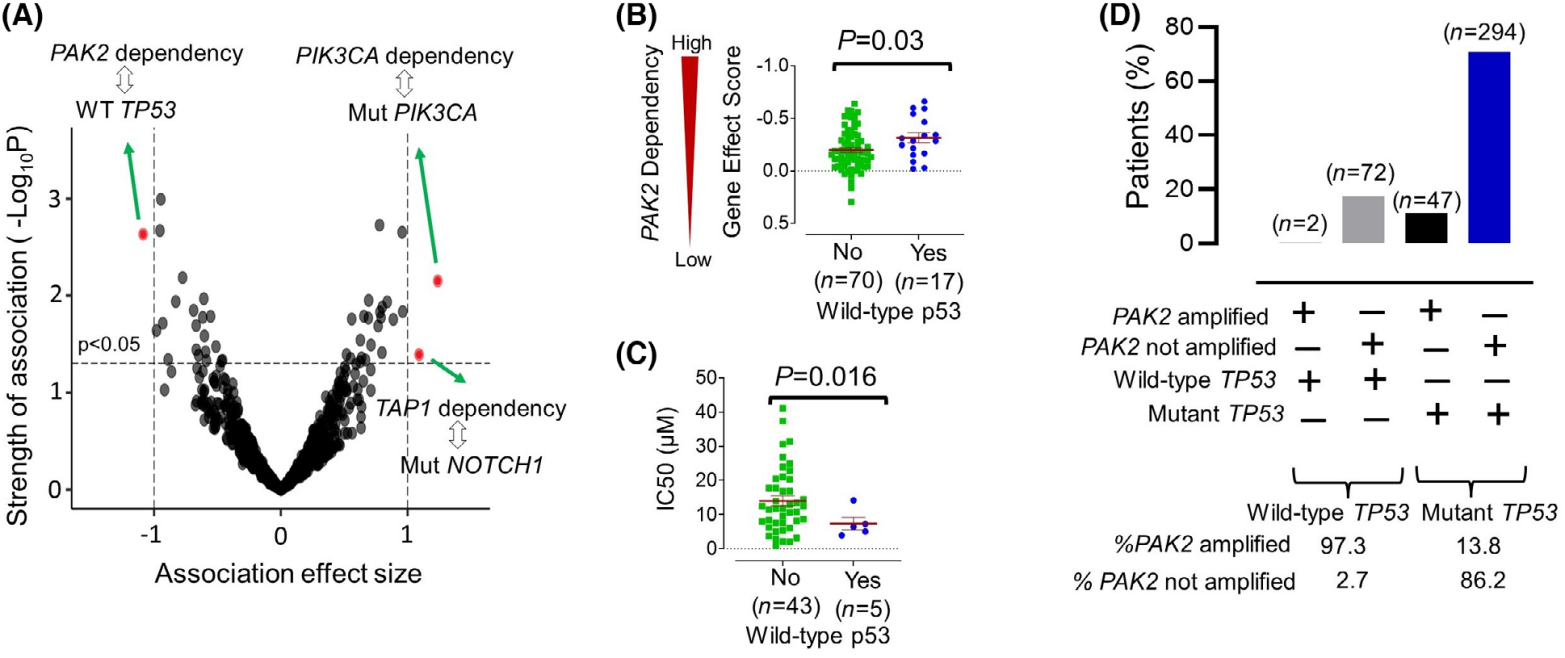
Association of PAK2 inhibitor responses with wild type *TP53*. (A) Evaluation of associations between median gene effect score of 143 prioritized targets and mutation status of *PIK3CA*, *CDKN2A*, *NOTCH1*, *FAT1* and *TP53* using Cohen's d effect size cutoff of ≥ 1 or ≤ −1 and significance cutoff of *P* < 0.05. (B) *TP53* mutation status vs. *PAK2* Gene effect score in DepMap. (C) *TP53* mutation status vs. PAK‐5339 inhibitor response in GDSC. *P* values were calculated by unpaired *t* test. (D) The 415 TCGA HPV(−) HNSCC tumours in TCGA subdivided by *TP53* mutation status and presence or absence of *PAK2* amplification.

To pursue additional predictors of inhibitor responses for PAK2 and other targets, we evaluated the 143 prioritized dependencies for association with the common cancer‐related gene mutations present in HPV(−) HNSCC. This analysis used the five genes with common hotspot and/or driver mutations in HPV(−) HNSCCs that were both identified by TCGA and were also present in ≥5% of cell line models in DepMap, specifically *PIK3CA*, *CDKN2A*, *NOTCH1*, *FAT1* and *TP53* (Table S3). Three significant associations were observed (Fig. 3A), including the anticipated association of oncogenic *PIK3CA* mutation with *PIK3CA* gene dependency. An association of *NOTCH1* loss of function mutation with *TAP1* dependency was present but of unclear significance given that the *TAP1* gene product's role in antigen presentation is not predicted to impact cancer cell line viability or growth in standard culture. More notably, an association between PAK2 dependency and wild‐type (WT) *TP53* status was detected based on comparison of the 20.6% of cell line models with WT *TP53* to the 80.4% with *TP53* hotspot and/or damaging mutations (Fig. 3B). The predicted increase in PAK2 inhibitor sensitivity in presence of WT *TP53* was confirmed by enhanced responses to PAK‐5339 observed in the 5 HPV(−) HNSCC models with WT *TP53* in GDSC data (Fig. 3C). Therefore, we explored whether *TP53* status might offer clinical utility in addition to *PAK2* copy number in predicting inhibitor responses by assessing the overlap between *PAK2* amplification and *TP53* mutation in the 415 HPV(−) HNSCCs in TCGA (Fig. 3D). This analysis shows that WT *TP53* alone may predict favourable PAK2 inhibitor responses in the 17.8% of HPV(−) HNSCCs in TCGA with WT *TP53* because almost all the WT cases (97.3%) contained a diploid *PAK2* locus and had reduced *PAK2* levels as a group relative to those with mutant *TP53* (Fig. S6). However, most HPV(−) HNSCCs (82.2%) harbour mutant *TP53*, and consideration of *PAK2* copy number appears relevant in them in order to predict responses to PAK2. Despite insufficient HNSCC cell line data to test the interaction between *TP53* and *PAK2* in determining PAK‐5339 sensitivity, these findings support considering the status of both gene loci in prediction of PAK2 inhibitor responses.

### 3.7 Re‐evaluation of the prioritization pipeline using an updated DepMap data release

**Fig. 4:**
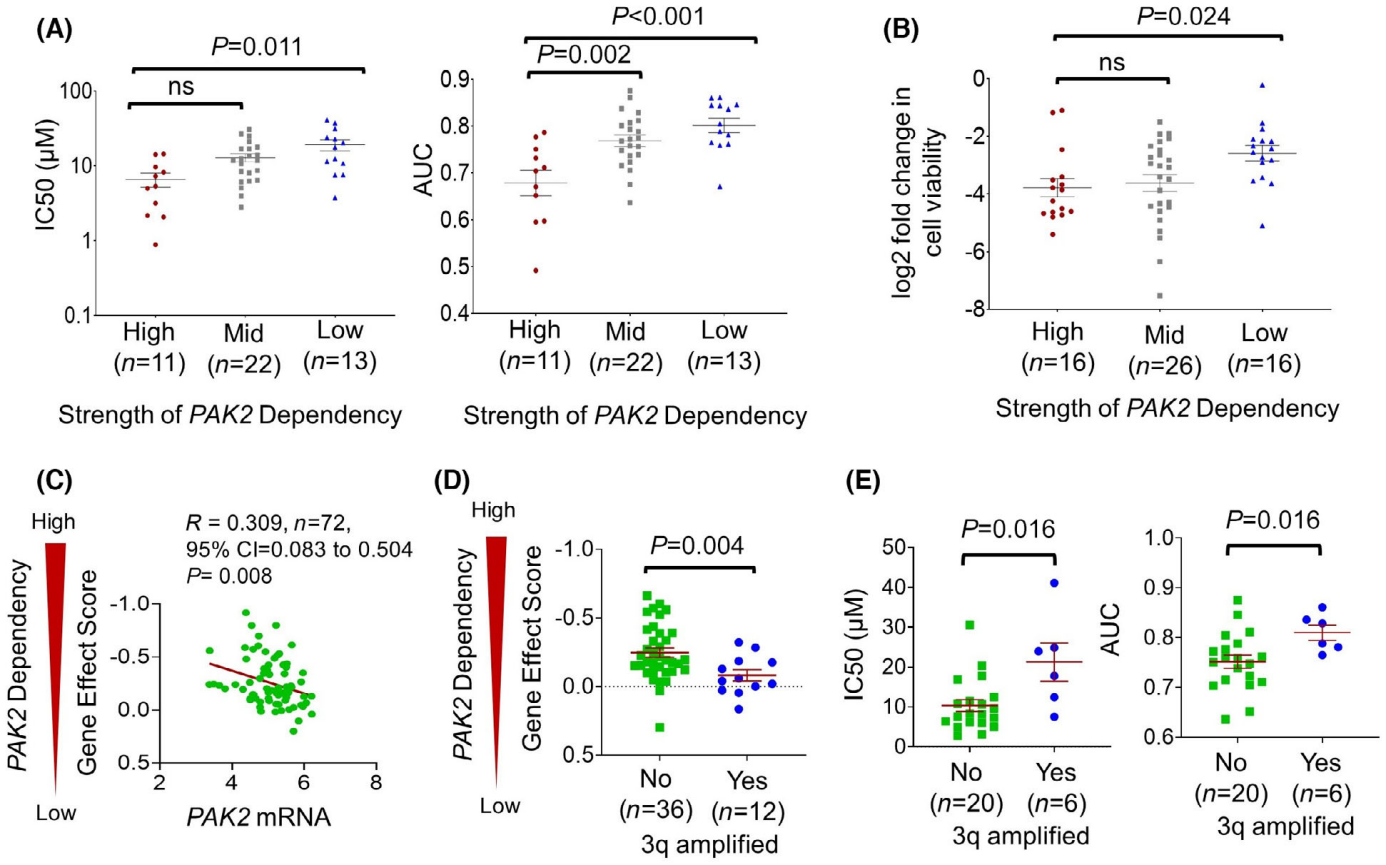
Confirmation of findings related to *PAK2* in the recent Depmap 23Q2 data release. (A) PAK‐5339 and (B) FRAX486 inhibitor responses *in vitro* in GDSC vs. strength of *PAK2* dependency in cell line models of HPV(−) HNSCC. High and low dependencies are defined by the top and bottom quartile of gene effect scores, respectively. Adjusted *P* values were defined by one‐way Welch's ANOVA corrected with Dunnett's multiple comparisons test. (C) *PAK2* dependency vs. mRNA expression in the cell line models. Pearson correlation coefficients were used to calculate *r* values, and *P* value was determined by *t* distribution. (D) *PAK2* dependency in cell lines with ABSOLUTE copy number data (*n* = 48) stratified by presence or absence of amplified 3q. (E) PAK‐5339 responses in subset of cell lines in (D) with inhibitor data in GDSC (*n* = 26). *P* values were calculated by Mann–Whitney *U* test.

To assess reproducibility of our findings in recently updated DepMap data prior to publication, the prioritization pipeline was reapplied to the 23Q2 data release. The pipeline prioritized 143 genes that included 103 genes in common with the previous prioritized list of 143 genes shown in Table S1. The new list is provided in Table S4, in which the 40 genes added to the list are highlighted and the 40 genes no longer meeting the dependency threshold are footnoted. Despite these changes, repeating functional analysis of the new prioritized gene list with the Gene Functional Classification Tool of DAVID Bioinformatics Resources (Table S5) yielded comparable results. Serine/threonine kinases including *PAK2* remained among the most enriched dependencies along with tyrosine kinases and RNA binding proteins. The previously identified mitochondrial carriers were regrouped into broader category of transmembrane receptors and carriers, and two new groups were comprised of immunoregulatory and unfolded protein response proteins. Evaluating the new list in the Open Targets Database for drugs reaching at least phase II identified 10 targets with inhibitors only studied clinically in other malignant and/or non‐malignant diseases (Table S6), including 7 preserved from the prior list (Table 2) and 3 additional agents. The relationship between *PAK2* gene effect scores and drug responses using both PAK‐5339 (Fig. 4A) and FRAX486 (Fig. 4B) was unchanged in the updated data release. Likewise, low mRNA levels of *PAK2* (Fig. 4C) and diploid status of the 3q amplicon (Fig. 4D) retained association with *PAK2* gene dependency, and the cell lines with diploid 3q exhibited enhanced drug responses (Fig. 4E). Lastly, a significant association was retained between PAK2 gene dependency and *TP53* WT status was retained (Fig. S7), although the effect size (−0.96) fell just short of the previous arbitrary effect size cutoff of −1 used in the analysis for 22Q1. These results show our key findings remained relatively stable using the updated DepMap 23Q2 release in spite of a ~ 33% change in the genes prioritized by our pipeline. Despite ongoing updating multiple public databases used in this study, this re‐analysis further underscores that the developed pipeline can easily be applied to subsequent data releases and other cancer types.

## 4 Discussion

This study used CRISPR screen data for HPV(−) HNSCC as a platform to create a pipeline for prioritizing new therapeutic targets in a manner generalizable to the other cancer types in DepMap. The *in vitro* CRISPR screens of cancer cell lines in DepMap inevitably miss gene dependencies that arise from the *in vivo* interface with the tumour microenvironment (e.g. PD1/PDL1). They are also predicted to miss targets derived from multiple genes with redundant function and those that can only provide anti‐tumour responses via combination therapies. Nevertheless, the efficacy of our pipeline is illustrated by its ability to capture the molecular targets for current HNSCC drugs including cetuximab, taxanes, and 5‐fluorouracil as well as genes for other well‐studied targets in HNSCC including other ErbB family members and PIK3CA. Favourable performance of the pipeline with these known targets supports the potential utility of other prioritized targets with clinical inhibitors that are well‐studied in other diseases and thus readily testable in HNSCC patients. Our prioritized list also reveals functional categories of genes that are much earlier in development as cancer therapy targets to be relevant to HPV(−) HNSCC. These include RNA‐binding proteins that regulate alternative mRNA splicing to serve cancer progression [39] and the SLC25 mitochondrial membrane nutrient transporters [37]. Immediate validation of hits in the screens was facilitated using existing inhibitor response data in GDSC. Although the current GDSC dataset is limited, we were able to validate the PAK2 serine–threonine kinase as a druggable dependency that is not well studied in HNSCC and to identify two potential genetic biomarkers of PAK2 inhibitor response. In comparing pipeline output between the 22Q1 and 23Q2 DepMap releases, it is noted that the updated dataset still prioritized *PAK2* and the same total number of genes but altered content of the gene list by 33%. The large change included addition of some new genes with fairly high gene effect scores, and the reason for this effect is not immediately apparent from the reported changes to the Chonos algorithm, which were focused on creating a 5% reduction in false positives meeting the dependency threshold. Nevertheless, we anticipate our pipeline will remain applicable to future updates that continue to refine the Chronos algorithm. Together, these findings provide a template for integration of the continuously updated DepMap CRISPR screen data with other emerging *in silico* resources to accelerate therapeutic development for both HNSCC and other cancer types.

PAK2 has both functional redundancies with the other group I PAK family members (PAK1 and 3) plus distinct roles that may jointly contribute to its utility as a target for HPV(−) HNSCC. The three group I PAK kinases all act downstream of Rho/Rac signalling [36] to activate β‐catenin [46] and other substrates involved in cell cycle progression (c‐myc, Raf, MEK1, LIM domain kinase), cytoskeletal dynamics (myosin light‐chain kinase, Merlin, β‐catenin), and apoptosis (CRAF, BAD) [36, 41, 46]. PAK2 also has distinct roles in inhibiting apoptosis by phosphorylating caspase 7 [47] and inhibiting caspase 3 activity [48], which may help explain why the knockout of *PAK2* but not *PAK1* or *PAK3* is embryonic lethal in mice [49]. Notably, the current group I PAK inhibitors (PAK‐5339, FRAX597, FRAX1036, and IPA‐3) are not PAK2 selective but have been shown experimentally to have direct antitumour effects [50, 51, 52] as well as ability to overcome chemoresistance [53, 54] in other cancer types.

On the surface, the associations of diploid *PAK2* plus low *PAK2* expression with *PAK2* gene dependence and favourable inhibitor responses seem paradoxical. One possible explanation is that deletion of all *PAK2* copies in CRISPR screens is less efficient in cell lines where *PAK2* is amplified. In addition, high *PAK2* expression in absence of PAK2 addiction might impede the efficacy of PAK‐5339. This scenario may arise if *PAK2* is amplified primarily as a passenger effect due to stronger selective pressure for copy gain in the adjacent oncogenes on the 3q amplicon. This situation appears related to the mechanism by which CYCLOPS (Copy‐number alterations Yielding Cancer Liabilities Owing to Partial losS) genes become favourable targets for cancer therapy [55]. The CYCLOPS phenomenon occurs when copy loss across a region containing a tumour suppressor decreases expression of an adjacent essential gene and thereby sensitizes to inhibition of the adjacent gene product. Despite absence of *PAK2* copy losses in HNSCC, lack of upregulated *PAK2* expression may improve inhibitor responses by similar mechanism in the 30% of HNSCCs without *PAK2* copy gain on 3q in TCGA.

The reasons for association of WT *TP53* status with *PAK2* dependency and favourable PAK2 inhibitor response also remain unclear. One possibility is that WT p53 provides protection from the genomic instability needed to amplify the 3q region containing *PAK2*. In addition, WT p53 positively regulates the promoters of two microRNAs, miR‐7‐5p [56] and miR‐455‐3p [57], that deplete the *PAK2* mRNA [58, 59] and thus could sensitize to inhibition by limiting PAK2 expression. Of note, the individual vs. combined effects of *TP53* status and *PAK2* copy number on inhibitor response could not be assessed here because too few cell lines had complete annotation for these genetic features in combination with PAK‐5339 inhibitor response data. Nevertheless, our results support making PAK2 a focus for clinical drug development and indicate that *TP53* mutation status and *PAK2* copy gains within the 3q amplicon should be jointly considered in future studies evaluating efficacy of PAK2 inhibition in HPV(−) HNSCC.

Another feature of our pipeline was incorporation of the Open Targets Database to prioritize targets with existing clinical inhibitors whose use for other diseases leaves them well positioned for clinical trial development in HPV(−) HNSCC. Another feature of our pipeline was incorporation of the Open Targets Database to prioritize targets with existing clinical inhibitors whose use for other diseases leaves them well positioned for clinical trial development in HPV(−) HNSCC. Among the 7 targets prioritized under both the 22Q1 and 23Q2 DepMap releases, there were two genes for targets with FDA‐approved agents that have been tested clinically in non‐malignant diseases but not in cancer (*UGCG*, *P2RY6*). Interestingly, the ceramide glucosyltransferase encoded by *UGCG*, which is involved in glycosphingolipid biosynthesis, has previously been linked to poor prognosis in HNSCC [60]. Another five targetable gene dependencies had clinical inhibitors that have reached at least a phase II trial (*BIRC2*, *ITGB1*, *MAP3K11*, *LDHA* and *PTPN1*), with 3 having been applied to other cancer types (*BIRC2*, *ITGB1*, and *LDHA*). These observations underscore the potential utility of our pipeline in guiding cancer type‐specific drug discovery and development in the near term.

## 5 Conclusion

This study catalogs the targetable gene dependencies that are most likely to be therapeutically relevant to HPV(−) HNSCC by integrating DepMAP CRISPR screen data with multiple other resources. Based on this analysis, several currently actionable targets for other diseases and multiple targets in earlier phases of development represent promising strategies for treating HNSCC. Of particular interest is *PAK2*, which was validated using existing PAK2 inhibitor response data and had identifiable genetic biomarkers of response. Our pipeline for HNSCC establishes a generalizable approach to filtering gene dependency data for other cancer types with a focus on accelerating therapeutic development.

## Conflict of interest

The authors declare no conflict of interest.

## Author contributions

DB, MKS, and ACC conceived the study, wrote the manuscript, and designed figures and tables. LS and PAG edited and provided critical feedback on the manuscript. MKS, ACC, RMB, PAG, PR, and LR performed bioinformatic and/or statistical analyses. All authors read and approved the final manuscript.

### Peer review

The peer review history for this article is available at https://publons.com/publon/10.1002/1878-0261.13558.

## Supporting information


**Table S1.** 143 prioritized targetable dependencies in cell line models of HNSCC.
**Table S2.** Prioritized targets with drugs in active or previous trials for HNSCC.
**Table S3.** Commonly mutated genes in HPV(−) HNSCC appearing in ≥ 5% of cell line models.
**Table S4.** Updated list of prioritized dependencies using DepMap 23Q2 data release, with added genes highlighted removed genes footnoted.
**Table S5.** Updated list of functional groups using DepMap 23Q2 data release, with added categories highlighted.
**Table S6.** Prioritized gene products with clinical inhibitors not well‐studied for HNSCC based on DepMap 23Q2 data release, with added targets highlighted removed targets highlighted.
**Fig. S1.** Cell line responses to inhibitors of prioritized targets already well‐studied in HNSCC.
**Fig. S2.** PAK‐5339 dose responses of HNSCC models in top vs. bottom quartile of *PAK2* gene effect score.
**Fig. S3.** *PAK2* dependency in HNSCC models based on CRISPR vs RNAi screening.
**Fig. S4.** Gene effect score distribution for *PAK2* vs. well‐studied targets in HNSCC.
**Fig. S5.** *PAK2* copy number alteration vs. survival of HPV(−) HNSCC patients in TCGA.
**Fig. S6.** *TP53* mutation status vs. PAK2 expression and copy number in HPV(−) HNSCCs in TCGA.
**Fig. S7.** Retained association of *PAK2* dependency with *TP53* WT status in 23Q2 data release.

## Data Availability

The data that support the findings of this study are available in Tables S1–S6 and Figs S1–S7. Gene dependency data processed by the Chronos algorithm was available from the DepMap at the Broad Institute at https://depmap.org/portal/. Data for the Head and Neck Squamous Cell Carcinoma TCGA cohort (project TCGA‐HNSCC, *n* = 523 cases) was available in the Genomic Data Commons via cBioPortal at https://www.cbioportal.org/.
