# Supplementary material for: A novel pipeline for prioritizing cancer type‐specific therapeutic vulnerabilities using DepMap identifies PAK2 as a target in head and neck squamous cell carcinomas
Source: Mol Oncol. 2023 Dec 13;18(2):336–49. doi: 10.1002/1878-0261.13558 (PMC10850805; doi:10.1002/1878-0261.13558)
Supplement: Supplementary file 1 — Table S1. 143 prioritized targetable dependencies in cell line models of HNSCC. Table S2. Prioritized targets with drugs in active or previous trials for HNSCC. Table S3. Commonly mutated genes in HPV(−) HNSCC appearing in ≥ 5% of cell line models. Table S4. Updated list of prioritized dependencies using DepMap 23Q2 data release, with added genes highlighted removed genes footnoted. Table S5. Updated list of functional groups using DepMap 23Q2 data release, with added categories highlighted. Table S6. Prioritized gene products with clinical inhibitors not well‐studied for HNSCC based on DepMap 23Q2 data release, with added targets highlighted removed targets highlighted. Fig. S1. Cell line responses to inhibitors of prioritized targets already well‐studied in HNSCC. Fig. S2. PAK‐5339 dose responses of HNSCC models in top vs. bottom quartile of PAK2 gene effect score. Fig. S3. PAK2 dependency in HNSCC models based on CRISPR vs RNAi screening. Fig. S4. Gene effect score distribution for PAK2 vs. well‐studied targets in HNSCC. Fig. S5. PAK2 copy number alteration vs. survival of HPV(−) HNSCC patients in TCGA. Fig. S6. TP53 mutation status vs. PAK2 expression and copy number in HPV(−) HNSCCs in TCGA. Fig. S7. Retained association of PAK2 dependency with TP53 WT status in 23Q2 data release. [file MOL2-18-336-s001.pdf]

SUPPLEMENTARY TABLES AND FIGURES:

Supplemental Table 1: 143 prioritized targetable dependencies in cell line models of HNSCC.

% Cell lines with essentiality	Median gene effect score	Gene symbol	Gene name
74	-0.76	*MBTPS1*	membrane bound transcription factor peptidase, site 1
71	-0.54	*KRT18*	keratin 18
70	-0.71	*CDK6*	cyclin dependent kinase 6
68	-0.59	*FARS2*	phenylalanyl-tRNA synthetase 2, mitochondrial
66	-0.6	*EGFR*	epidermal growth factor receptor
60	-0.57	*PIK3CA*	phosphatidylinositol-4,5-bisphosphate 3-kinase catalytic subunit alpha
59	-0.89	*TYMS*	thymidylate synthetase
57	-0.56	*KLF5*	Kruppel like factor 5
56	-0.68	*TUBB4B*	tubulin beta 4B class IVb
54	-0.55	*UBA6*	ubiquitin like modifier activating enzyme 6
53	-0.54	*RBM10*	RNA binding motif protein 10
52	-0.55	*CARM1*	coactivator associated arginine methyltransferase 1
52	-0.53	*NRDC*	nardilysin convertase
51	-0.67	*SLC2A1*	solute carrier family 2 member 1
51	-0.59	*CFLAR*	CASP8 and FADD like apoptosis regulator
51	-0.52	*SSR2*	signal sequence receptor subunit 2
48	-0.6	*TP63*	tumor protein p63
48	-0.56	*ITGB1*	integrin subunit beta 1
47	-0.49	*EARS2*	glutamyl-tRNA synthetase 2, mitochondrial
46	-0.47	*HNRNPA1*	heterogeneous nuclear ribonucleoprotein A1
45	-0.59	*ERBB2*	erb-b2 receptor tyrosine kinase 2
43	-0.56	*PPIH*	peptidylprolyl isomerase H
43	-0.52	*HSD17B12*	hydroxysteroid 17-beta dehydrogenase 12
43	-0.48	*SYT15*	synaptotagmin 15
41	-0.55	*ERBB3*	erb-b2 receptor tyrosine kinase 3
41	-0.48	*ASH2L*	ASH2 like, histone lysine methyltransferase complex subunit
41	-0.47	*PMS2*	PMS1 homolog 2, mismatch repair system component
40	-0.67	*DHRSX*	dehydrogenase/reductase X-linked
40	-0.53	*PTBP1*	polypyrimidine tract binding protein 1
40	-0.48	*SLC3A2*	solute carrier family 3 member 2
39	-0.51	*ODR4*	odr-4 GPCR localization factor homolog
38	-0.68	*TIPARP*	TCDD inducible poly(ADP-ribose) polymerase
38	-0.49	*EXTL2*	exostosin like glycosyltransferase 2
37	-0.58	*TMX2*	thioredoxin related transmembrane protein 2
37	-0.54	*SLC4A7*	solute carrier family 4 member 7
37	-0.49	*PTP4A1*	protein tyrosine phosphatase 4A1

37	-0.47	*ZRANB2*	zinc finger RANBP2-type containing 2
36	-0.82	*GPX4*	glutathione peroxidase 4
36	-0.58	*PKN2*	protein kinase N2
34	-0.5	*NDUFS3*	NADH:ubiquinone oxidoreductase core subunit S3
34	-0.48	*SLC4A5*	solute carrier family 4 member 5
33	-0.49	*CHMP1A*	charged multivesicular body protein 1A
32	-0.53	*PPME1*	protein phosphatase methylesterase 1
31	-0.47	*NISCH*	nischarin
30	-0.49	*BOLA3*	bolA family member 3
30	-0.45	*KCNJ9*	potassium inwardly rectifying channel subfamily J member 9
29	-0.55	*MARK2*	microtubule affinity regulating kinase 2
29	-0.5	*BIRC2*	baculoviral IAP repeat containing 2
28	-0.54	*SPNS1*	sphingolipid transporter 1 (putative)
28	-0.46	*CSF3*	colony stimulating factor 3
26	-0.51	*KCNA10*	potassium voltage-gated channel subfamily A member 10
26	-0.49	*TXNDC9*	thioredoxin domain containing 9
26	-0.49	*LCN1*	lipocalin 1
26	-0.45	*ADAM11*	ADAM metallopeptidase domain 11
25	-0.61	*MAP3K11*	mitogen-activated protein kinase kinase kinase 11
25	-0.5	*RIPK3*	receptor interacting serine/threonine kinase 3
25	-0.44	*ABCA4*	ATP binding cassette subfamily A member 4
25	-0.43	*ENDOG*	endonuclease G
25	-0.43	*INS*	insulin
24	-0.52	*PAK2*	p21 (RAC1) activated kinase 2
24	-0.47	*PROCA1*	protein interacting with cyclin A1
24	-0.46	*H3C4*	H3 clustered histone 4
23	-0.51	*CYB5R4*	cytochrome b5 reductase 4
23	-0.49	*TYRO3*	TYRO3 protein tyrosine kinase
23	-0.48	*CNTNAP3B*	contactin associated protein family member 3B
22	-0.62	*RAB35*	RAB35, member RAS oncogene family
22	-0.49	*IMPDH1*	inosine monophosphate dehydrogenase 1
22	-0.49	*ELAVL1*	ELAV like RNA binding protein 1
22	-0.48	*RNF31*	ring finger protein 31
22	-0.48	*PI4KB*	phosphatidylinositol 4-kinase beta
22	-0.48	*GRK2*	G protein-coupled receptor kinase 2
20	-0.56	*PDE12*	phosphodiesterase 12
20	-0.52	*ITGA3*	integrin subunit alpha 3
20	-0.46	*PTAR1*	protein prenyltransferase alpha subunit repeat containing 1
20	-0.45	*FASN*	fatty acid synthase
20	-0.45	*ARTN*	artemin
18	-0.47	*KCNK7*	potassium two pore domain channel subfamily K member 7
17	-0.48	*ADAMTSL4*	ADAMTS like 4
17	-0.47	*CYP4F11*	cytochrome P450 family 4 subfamily F member 11
17	-0.46	*SCGB2A1*	secretoglobin family 2A member 1
17	-0.45	*SLC29A2*	solute carrier family 29 member 2

17	-0.43	*LTF*	lactotransferrin
16	-0.62	*TDP2*	tyrosyl-DNA phosphodiesterase 2
16	-0.47	*SLC25A33*	solute carrier family 25 member 33
16	-0.46	*FGF19*	fibroblast growth factor 19
16	-0.45	*SPINT1*	serine peptidase inhibitor, Kunitz type 1
16	-0.43	*H4C5*	H4 clustered histone 5
16	-0.39	*TAP1*	transporter 1, ATP binding cassette subfamily B member
15	-0.49	*EP300*	E1A binding protein p300
15	-0.49	*UGCG*	UDP-glucose ceramide glucosyltransferase
15	-0.46	*LDLR*	low density lipoprotein receptor
15	-0.45	*MAP4K2*	mitogen-activated protein kinase kinase kinase kinase 2
15	-0.44	*MANF*	mesencephalic astrocyte derived neurotrophic factor
15	-0.44	*SLC25A1*	solute carrier family 25 member 1
15	-0.38	*CYP2A13*	cytochrome P450 family 2 subfamily A member 13
14	-0.54	*IGF1R*	insulin like growth factor 1 receptor
14	-0.52	*SLC7A1*	solute carrier family 7 member 1
14	-0.5	*CHST8*	carbohydrate sulfotransferase 8
14	-0.48	*LMNA*	lamin A/C
14	-0.48	*OVGP1*	oviductal glycoprotein 1
14	-0.47	*GUCA2A*	guanylate cyclase activator 2A
14	-0.46	*RPS6KB1*	ribosomal protein S6 kinase B1
14	-0.46	*HCRTR1*	hypocretin receptor 1
14	-0.45	*LGALS9*	galectin 9
14	-0.45	*LIG1*	DNA ligase 1
14	-0.42	*ITM2B*	integral membrane protein 2B
13	-0.52	*TM2D1*	TM2 domain containing 1
13	-0.51	*NAGLU*	N-acetyl-alpha-glucosaminidase
13	-0.48	*HTRA2*	HtrA serine peptidase 2
13	-0.46	*CDK8*	cyclin dependent kinase 8
13	-0.46	*EIF4H*	eukaryotic translation initiation factor 4H
13	-0.43	*HNRNPUL2*	heterogeneous nuclear ribonucleoprotein U like 2
13	-0.43	*ABCB10*	ATP binding cassette subfamily B member 10
13	-0.39	*ADAMTS7*	ADAM metallopeptidase with thrombospondin type 1 motif 7
11	-0.53	*LTB4R2*	leukotriene B4 receptor 2
11	-0.5	*P2RY6*	pyrimidinergic receptor P2Y6
11	-0.49	*AHCYL1*	adenosylhomocysteinase like 1
11	-0.49	*RNF123*	ring finger protein 123
11	-0.48	*PCSK9*	proprotein convertase subtilisin/kexin type 9
11	-0.47	*RBM5*	RNA binding motif protein 5
11	-0.47	*LDHA*	lactate dehydrogenase A
11	-0.45	*HAO2*	hydroxyacid oxidase 2
11	-0.44	*SLC22A25*	solute carrier family 22 member 25
11	-0.43	*LTBP3*	latent transforming growth factor beta binding protein 3
11	-0.41	*PTPN1*	protein tyrosine phosphatase non-receptor type 1
10	-0.52	*GSTM3*	glutathione S-transferase mu 3

10	-0.5	*OGA*	O-GlcNAcase
10	-0.45	*RTN3*	reticulon 3
10	-0.45	*GANAB*	glucosidase II alpha subunit
10	-0.44	*RTN4IP1*	reticulon 4 interacting protein 1
10	-0.43	*GGTLC1*	gamma-glutamyltransferase light chain 1
10	-0.41	*TNFRSF8*	TNF receptor superfamily member 8
9	-0.51	*EGLN1*	egl-9 family hypoxia inducible factor 1
9	-0.46	*PRKACA*	protein kinase cAMP-activated catalytic subunit alpha
9	-0.46	*ASIC1*	acid sensing ion channel subunit 1
9	-0.46	*CCL7*	C-C motif chemokine ligand 7
9	-0.46	*TAC4*	tachykinin precursor 4
9	-0.45	*CDC25B*	cell division cycle 25B
9	-0.43	*MRGPRX3*	MAS related GPR family member X3
9	-0.42	*CCS*	copper chaperone for superoxide dismutase
9	-0.42	*RPS6KA4*	ribosomal protein S6 kinase A4
9	-0.76	*MTCH2*	mitochondrial carrier 2
9	-0.54	*SLC25A25*	solute carrier family 25 member 25

Supplemental Table 2: Prioritized targets with drugs in active or previous trials for HNSCC.

Gene symbol	Gene name	Most advanced status of inhibitor	Approved indications	Approved agents	Phase II/III trials for other diseases	Phase II/III agents
EGFR	Epidermal growth factor receptor	Approved	Head and neck cancer, non-small cell lung cancer, breast cancer and other malignant diseases	Cetuximab, Osimertinib, Afatinib, Vandetanib	Urothelial carcinoma, cervical cancer, soft tissue sarcoma	Panitumumab, Nimotuzumab, Lapatinib
TYMS	Thymidylate synthetase	Approved	Head and neck cancer, breast cancer, lung adenocarcinoma, and other malignant diseases	Capecitabine, Pemetrexed, Fluorouracil	Gastric cancer, pancreatic cancer, hepatocellular carcinoma	Tegafur, Doxifluridine
TUBB4B	Tubulin beta 4B class IVb	Approved	Head and neck cancer, Breast cancer, prostate adenocarcinoma, and other malignant diseases	Ixabepilone, Docetaxel, Paclitaxel, Eribulin	-	-
PIK3CA	Phosphatidylinositol-4,5-bisphosphate 3-kinase catalytic subunit alpha	Approved	Breast cancer, follicular lymphoma	Alpelisib, Copanlisib	Head and neck cancer, non-Hodgkins lymphoma, ovarian cancer and other malignant diseases	Dactolisib, Buparlisib, Taselisib, Copanlisib
ERBB2	erb-b2 receptor tyrosine kinase 2	Approved	Breast cancer, non-small cell lung carcinoma, thyroid carcinoma, neoplasm	Pertuzumab, Pyrotinib, Vandetanib, Pertuzumab, Trastuzumab	Head and neck cancer, colorectal cancer, and other malignant diseases	
ERBB3	Erb-b2 receptor tyrosine kinase 3	Approved	Thyroid carcinoma	Vandetanib	non-small cell lung carcinoma, Head and neck cancer and other malignant diseases	Patritumab
CDK6	Cyclin dependent kinase 6	Approved	Breast cancer, small cell lung carcinoma	Ribociclib, Palbociclib, Trilaciclib, Abemaciclib	Head and neck cancer, soft tissue sarcoma and other malignant diseases	
IGF1R	Insulin like growth factor 1 receptor	Approved	Graves ophthalmopathy, hypothyroidism	Teprotumumab, Mecasermin	Head and neck cancer, Breast cancer, prostate cancer, colorectal cancer and other malignant diseases	Cixutumumab, Linsitinib, AXL-1717
NDUFS3	NADH:ubiquinone oxidoreductase core subunit S3	Approved	Mouth neoplasm, diabetes mellitus, metabolic disorder and other non-malignant diseases	Metformin	Liver cancer, thyroid cancer, prostate cancer, head and neck cancer and other malignant diseases	-

Supplementary Table 3: Commonly mutated genes in HPV(-) HNSCC appearing in ≥5% of cell line models.

Gene name	Putative role	% with mutation in TCGA	% Cell lines with mutation
PIK3CA	oncogene	15.9	12.6
CDKN2A	tumor suppressor	24.6	25.3
NOTCH1	tumor suppressor	19.5	8
FAT1	mixed	25.8	20.7
TP53	mixed	82.2	80.5

Supplemental Table 4: Updated list of prioritized dependencies using DepMap 23Q2 data release, with added genes highlighted removed genes footnoted[1].

% Cell Lines	Median Gene Effect	Gene Symbol	Gene Name
75	-0.95	*PPIA*	peptidylprolyl isomerase A
70	-0.91	*CDK6*	cyclin dependent kinase 6
67	-1.1	*TUBA1C*	tubulin alpha 1c
67	-0.73	*TUBA3D*	tubulin alpha 3d
63	-0.92	*TYMS*	thymidylate synthetase
63	-0.75	*EGFR*	epidermal growth factor receptor
62	-0.83	*MBTPS1*	membrane bound transcription factor peptidase, site 1
62	-0.64	*UBA6*	ubiquitin like modifier activating enzyme 6
61	-0.73	*KLF5*	Kruppel like factor 5
61	-0.62	*OR1D2*	olfactory receptor family 1 subfamily D member 2
56	-0.87	*TUBB4B*	tubulin beta 4B class IVb
56	-0.59	*OR7A10*	olfactory receptor family 7 subfamily A member 10
54	-0.62	*PIK3CA*	phosphatidylinositol-4,5-bisphosphate 3-kinase catalytic subunit alpha
53	-0.87	*FCGR1A*	Fc gamma receptor Ia
53	-0.65	*RBM10*	RNA binding motif protein 10
53	-0.64	*FARS2*	phenylalanyl-tRNA synthetase 2, mitochondrial
51	-0.84	*SLC2A1*	solute carrier family 2 member 1
46	-0.69	*EARS2*	glutamyl-tRNA synthetase 2, mitochondrial
46	-0.56	*OR4A47*	olfactory receptor family 4 subfamily A member 47
45	-0.81	*TP63*	tumor protein p63
45	-0.71	*ERBB2*	erb-b2 receptor tyrosine kinase 2
45	-0.62	*PKN2*	protein kinase N2
45	-0.58	*HNRNPA1*	heterogeneous nuclear ribonucleoprotein A1
44	-0.77	*ITGB1*	integrin subunit beta 1
44	-0.56	*LDHA*	lactate dehydrogenase A
43	-0.76	*CFLAR*	CASP8 and FADD like apoptosis regulator
43	-0.72	*TIPARP*	TCDD inducible poly(ADP-ribose) polymerase
40	-0.72	*SLC4A7*	solute carrier family 4 member 7
40	-0.65	*ERBB3*	erb-b2 receptor tyrosine kinase 3
39	-0.63	*HSD17B12*	hydroxysteroid 17-beta dehydrogenase 12
39	-0.57	*NISCH*	Nischarin
39	-0.55	*CARM1*	coactivator associated arginine methyltransferase 1
39	-0.54	*LGALS9*	galectin 9
38	-0.61	*OR7A17*	olfactory receptor family 7 subfamily A member 17
37	-0.56	*NRDC*	nardilysin convertase
37	-0.54	*AMY2A*	amylase alpha 2A
34	-0.63	*PTBP1*	polypyrimidine tract binding protein 1
34	-0.53	*SFTA2*	surfactant associated 2

[1] **Removed genes:** *KRT18, SYT15, PMS2, DHRSX, CSF3, LCN1, ABCA4, CNTNAP3B, IMPDH1, ARTN, ADAMTSL4, CYP4F11, SLC25A33, H4C5, TAP1, CYP2A13, RPS6KB1, HCRTR1, ITM2B, NAGLU, EIF4H, ABCB10, ADAMTS7, LTB4R2, PCSK9, SLC22A25, LTBP3, OGA, RTN3, GANAB, TNFRSF8, EGLN1, PRKACA, ASIC1, CCL7, TAC4, CCS, RPS6KA4, MTCH2, SLC25A25*

33	-0.76	*MARK2*	microtubule affinity regulating kinase 2
33	-0.55	*SPNS1*	sphingolipid transporter 1 (putative)
33	-0.54	*INS*	Insulin
32	-0.56	*ZRANB2*	zinc finger RANBP2-type containing 2
32	-0.54	*BOLA3*	bolA family member 3
31	-0.63	*PPIH*	peptidylprolyl isomerase H
31	-0.62	*OR4C46*	olfactory receptor family 4 subfamily C member 46
31	-0.52	*RNF123*	ring finger protein 123
30	-0.85	*GPX4*	glutathione peroxidase 4
30	-0.58	*PPME1*	protein phosphatase methylesterase 1
30	-0.56	*PTP4A1*	protein tyrosine phosphatase 4A1
29	-0.54	*OR1E2*	olfactory receptor family 1 subfamily E member 2
28	-0.58	*SSR2*	signal sequence receptor subunit 2
26	-0.62	*BIRC2*	baculoviral IAP repeat containing 2
25	-0.59	*ODR4*	odr-4 GPCR localization factor homolog
25	-0.56	*GRK2*	G protein-coupled receptor kinase 2
25	-0.55	*SLC3A2*	solute carrier family 3 member 2
24	-0.6	*TMX2*	thioredoxin related transmembrane protein 2
24	-0.55	*H3C4*	H3 clustered histone 4
23	-0.59	*OR4K1*	olfactory receptor family 4 subfamily K member 1
23	-0.57	*CSH2*	chorionic somatomammotropin hormone 2
23	-0.57	*KCNJ9*	potassium inwardly rectifying channel subfamily J member 9
22	-0.62	*FASN*	fatty acid synthase
22	-0.56	*ASH2L*	ASH2 like, histone lysine methyltransferase complex subunit
22	-0.55	*ADAM11*	ADAM metallopeptidase domain 11
21	-0.64	*PDE12*	phosphodiesterase 12
21	-0.61	*MAP3K11*	mitogen-activated protein kinase kinase kinase 11
21	-0.58	*OR4N2*	olfactory receptor family 4 subfamily N member 2
21	-0.52	*PROCA1*	protein interacting with cyclin A1
20	-0.72	*RAB35*	RAB35, member RAS oncogene family
20	-0.59	*UGCG*	UDP-glucose ceramide glucosyltransferase
18	-0.6	*ITGA3*	integrin subunit alpha 3
18	-0.57	*LMNA*	lamin A/C
18	-0.57	*EXTL2*	exostosin like glycosyltransferase 2
18	-0.56	*TYRO3*	TYRO3 protein tyrosine kinase
18	-0.51	*OR6C6*	olfactory receptor family 6 subfamily C member 6
18	-0.49	*CD276*	CD276 molecule
17	-0.62	*TXNDC9*	thioredoxin domain containing 9
17	-0.6	*SNAP29*	synaptosome associated protein 29
17	-0.57	*PAK2*	p21 (RAC1) activated kinase 2
17	-0.56	*CXADR*	CXADR Ig-like cell adhesion molecule
17	-0.56	*PTAR1*	protein prenyltransferase alpha subunit repeat containing 1
17	-0.56	*RNF31*	ring finger protein 31
17	-0.53	*SLC4A5*	solute carrier family 4 member 5
16	-0.56	*TM2D1*	TM2 domain containing 1

16	-0.55	*TRIM43*	tripartite motif containing 43
16	-0.54	*OR7G2*	olfactory receptor family 7 subfamily G member 2
16	-0.5	*SLC25A1*	solute carrier family 25 member 1
15	-0.65	*TDP2*	tyrosyl-DNA phosphodiesterase 2
15	-0.58	*NPB*	neuropeptide B
15	-0.56	*CYB5R4*	cytochrome b5 reductase 4
15	-0.56	*RTN4IP1*	reticulon 4 interacting protein 1
15	-0.56	*SLC7A1*	solute carrier family 7 member 1
15	-0.55	*MAP4K2*	mitogen-activated protein kinase kinase kinase kinase 2
15	-0.54	*HNRNPUL2*	heterogeneous nuclear ribonucleoprotein U like 2
14	-0.63	*HTRA2*	HtrA serine peptidase 2
14	-0.63	*IGF1R*	insulin like growth factor 1 receptor
14	-0.58	*PTPN1*	protein tyrosine phosphatase non-receptor type 1
14	-0.56	*NDUFS3*	NADH:ubiquinone oxidoreductase core subunit S3
14	-0.55	*PI4KB*	phosphatidylinositol 4-kinase beta
14	-0.53	*CD151*	CD151 molecule (Raph blood group)
14	-0.53	*GGTLC1*	gamma-glutamyltransferase light chain 1
14	-0.47	*CXCL2*	C-X-C motif chemokine ligand 2
13	-0.59	*ELAVL1*	ELAV like RNA binding protein 1
13	-0.57	*KCNK7*	potassium two pore domain channel subfamily K member 7
13	-0.57	*LTF*	Lactotransferrin
13	-0.56	*FGF19*	fibroblast growth factor 19
13	-0.56	*HAO2*	hydroxyacid oxidase 2
13	-0.52	*RIPK3*	receptor interacting serine/threonine kinase 3
13	-0.52	*CDK8*	cyclin dependent kinase 8
11	-0.67	*EP300*	E1A binding protein p300
11	-0.64	*IKBKG*	inhibitor of nuclear factor kappa B kinase regulatory subunit gamma
11	-0.58	*CDC25B*	cell division cycle 25B
11	-0.57	*OVGP1*	oviductal glycoprotein 1
11	-0.56	*RBM5*	RNA binding motif protein 5
11	-0.55	*PYGM*	glycogen phosphorylase, muscle associated
11	-0.54	*SPINT1*	serine peptidase inhibitor, Kunitz type 1
11	-0.53	*CHST8*	carbohydrate sulfotransferase 8
11	-0.52	*OR10A4*	olfactory receptor family 10 subfamily A member 4
11	-0.5	*OR4D9*	olfactory receptor family 4 subfamily D member 9
10	-0.64	*KCNA10*	potassium voltage-gated channel subfamily A member 10
10	-0.61	*LDLR*	low density lipoprotein receptor
10	-0.6	*AHCYL1*	adenosylhomocysteinase like 1
10	-0.59	*GSTM3*	glutathione S-transferase mu 3
10	-0.59	*APH1A*	aph-1 homolog A, gamma-secretase subunit
10	-0.59	*GUCA2A*	guanylate cyclase activator 2A
10	-0.57	*MANF*	mesencephalic astrocyte derived neurotrophic factor
10	-0.55	*ENDOG*	endonuclease G
10	-0.54	*PTP4A2*	protein tyrosine phosphatase 4A2
10	-0.5	*RCE1*	Ras converting CAAX endopeptidase 1

10	-0.49	*PDK2*	pyruvate dehydrogenase kinase 2
9	-0.65	*TGFBR1*	transforming growth factor beta receptor 1
9	-0.62	*P2RY6*	pyrimidinergic receptor P2Y6
9	-0.61	*YWHAG*	tyrosine 3-monooxygenase/tryptophan 5-monooxygenase activation protein gamma
9	-0.61	*DERL1*	derlin 1
9	-0.6	*CHMP1A*	charged multivesicular body protein 1A
9	-0.59	*SLC25A51*	solute carrier family 25 member 51
9	-0.55	*LIG1*	DNA ligase 1
9	-0.52	*SCGB2A1*	secretoglobin family 2A member 1
9	-0.51	*CXCL1*	C-X-C motif chemokine ligand 1
9	-0.51	*SLC29A2*	solute carrier family 29-member 2
9	-0.51	*MAST1*	microtubule associated serine/threonine kinase 1
9	-0.49	*TRIM46*	tripartite motif containing 46
9	-0.49	*MRGPRX3*	MAS related GPR family member X3
9	-0.49	*MATN1*	matrilin 1

Supplementary Table 5: Updated list of functional groups using DepMap 23Q2 data release, with added categories highlighted.

**Functional classification**	**Enrichment score**	**Genes (Median gene effect score, percentage cell lines)**
Serine/threonine kinases	2.84	*MARK2* (-0.76, 33%), *PKN2* (-0.57, 17%), *MAP4K2* (-0.55, 15%), *MAP3K11* (-0.61, 21%), *MAST1* (-0.51, 9), *PAK2* (-0.57, 17%), *CDK8* (-0.52, 13%)
Tyrosine kinases	2.83	*EGFR* (-0.75, 63%), *TYRO3* (-0.56, 18%), *ERBB3* (-0.65, 40%), *ERBB2* (-0.71, 45%), *IGF1R* (-0.63, 14%)
RNA-binding proteins	0.96	*HNRNPA1* (-0.58, 45%), *RBM5* (-0.56, 11%), *PTBP1* (-0.63, 34%), *ELAVL1* (-0.59, 13%), *RBM10* (-0.65, 53%), *ZRANB2* (-0.56, 32%)
Transmembrane receptors and carriers	2.72	*TM2D1* (-0.56, 16%), *SLC25A1* (-0.5, 16%), *SPNS1* (-0.55, 33%), *OR4C46 (-*0.62, 31%), *OR7A10* (-0.59, 56%), *OR4A47* (-0.56, 46%), *CD151* (-0.53, 14%), *SLC25A51* (-0.59, 9%), *KCNK7* (-0.57, 13%), *KCNA10* (-0.64, 10%), *OR7G2* (-0.54, 16%), *OR10A4* (-0.52, 11%), *SSR2* (-0.58, 28%), *MRGPRX3* (-0.49, 9%), *OR1E2 (-0.54, 29%), OR4K1* (-0.59, 23%), *APH1A* (-0.59, 10%), *OR7A17* (-0.61, 38%), *ODR4* (-0.59, 25%), *OR1D2* (-0.62, 61%), *SLC7A1* (-0.56, 15%), *OR4D9* (-0.5, 11%), *KCNJ9* (-0.57, 23%), *P2RY6* (-0.62, 9%), *OR6C6* (-0.51, 18%), *ADAM11* (-0.55, 22%), *OR4N2* (-0.58, 21%)
Unfolded protein response	1.72	*MBTPS1* (-0.83, 62%*), CHST8* (-0.53, 11%), *SSR2* (-0.58, 28%), *TMX2* (-0.6, 24%), *EXTL2* (-0.57, 18%), *TM2D1* (-0.56, 16%)
Immunoregulatory Interactions	1.45	*FCGR1A* (-0.87, 53%), *CXADR* (-0.56, 17%), *CD276* (-0.49, 18%)

Supplementary Table 6: Prioritized gene products with clinical inhibitors not well-studied for HNSCC based on DepMap 23Q2 data release, with added targets highlighted and removed targets footnoted[2].

Gene symbol	Gene name	Most advanced status of inhibitor	Approved indications	Approved agents	Phase II/III trials for other diseases	Phase II/III agents
UGCG	UDP-glucose ceramide glucosyltransferase	Approved	Niemann-Pick disease, Type I Gaucher disease	Miglustat, Eliglustat	Other glycogen storage diseases, cystic fibrosis, HIV	Lucerastat
PPIA	Peptidylprolyl isomerase A	Approved	Dry eye syndrome, Kidney infection, Rheumatoid arthritis	Cyclosporine		
AMY2A	Amylase alpha 2A	Approved	Diabetes mellitus	Acarbose		
P2RY6	pyrimidinergic receptor P2Y6	#Approved	Dry eye syndrome	Diquafosol		
BIRC2	baculoviral IAP repeat containing 2	Phase II	-	-	Ovarian cancer	Birinapant
ITGB1	integrin subunit beta 1	Phase II	-	-	Renal cell carcinoma, pancreatic carcinoma, melanoma, NSCLC	Volociximab, ATN-161
MAP3K11	mitogen-activated protein kinase kinase kinase 11	Phase II	-	-	Parkinson disease	CEP-1347
LDHA	lactate dehydrogenase A	Phase II	-	-	NSCLC, adrenal cortical carcinoma, hyperoxaluria	Nedosiran, AT-101 (Gossypol)
PTPN1	protein tyrosine phosphatase non-receptor type 1	Phase II	-	-	Type 2 diabetes	Ertiprotafib, Trodusquemine
APH1A	Aph-1 homolog A, gamma-secretase subunit	Phase II			Alzheimer disease, desmoid tumor	Semagacestat, Tarenflurbil, Nirogacestat

[2] **Removed genes**: *PCSK9, IMPDH1, HCRTR1, GANAB, TNFRSF8, EGLN1, LTB4R2*

Supplementary Figure 1:

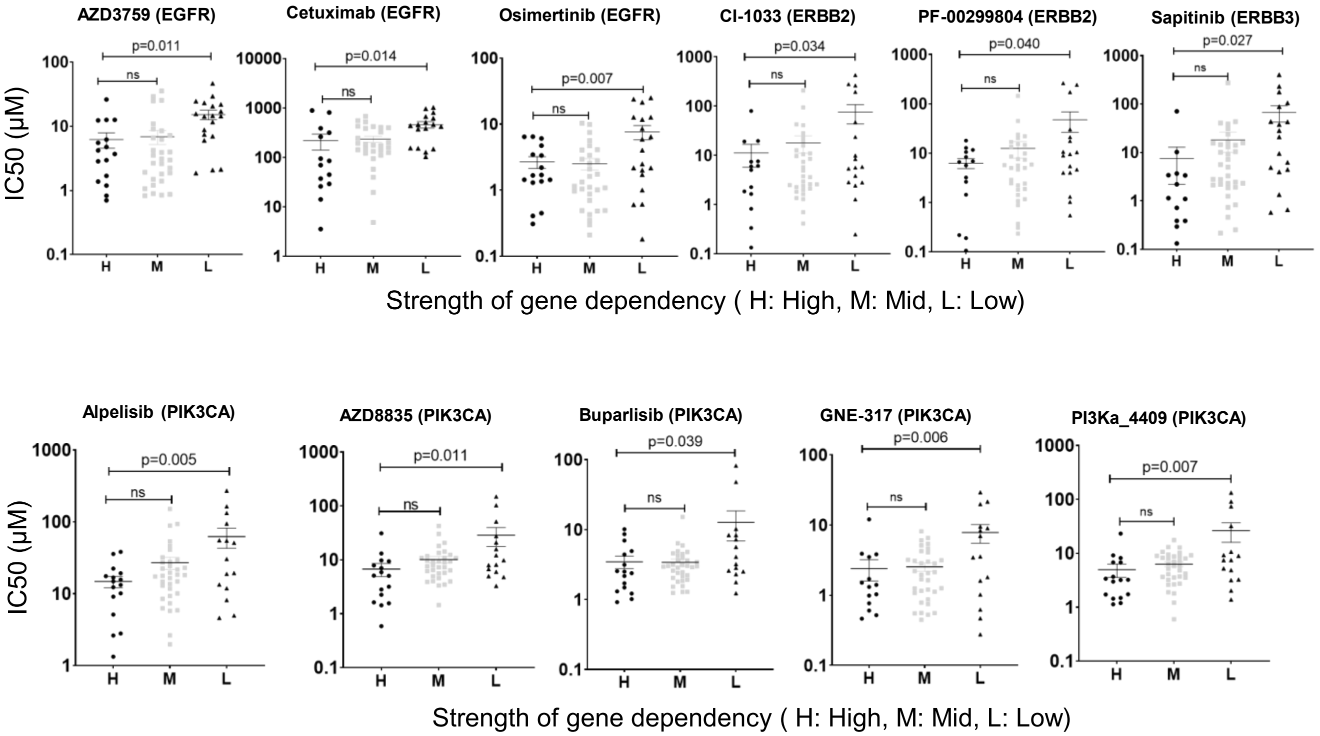

Supplementary Figure 1: **Cell line responses to inhibitors of prioritized targets already well-studied in HNSCC.** Inhibitor responses against EGFR, ERBB2, ERBB3, and PIK3CA *in vitro* in GDSC vs. strength of their dependency in cell line models of HPV(-) HNSCC. High and low dependencies are defined by the top and bottom quartile of gene effect scores, respectively. Adjusted p values were defined by one-way Welch's ANOVA corrected with Dunnett's multiple comparisons test. Cell viability was determined by Promega CellTiter-Glo^{TM} assay.

Supplementary Figure 2:

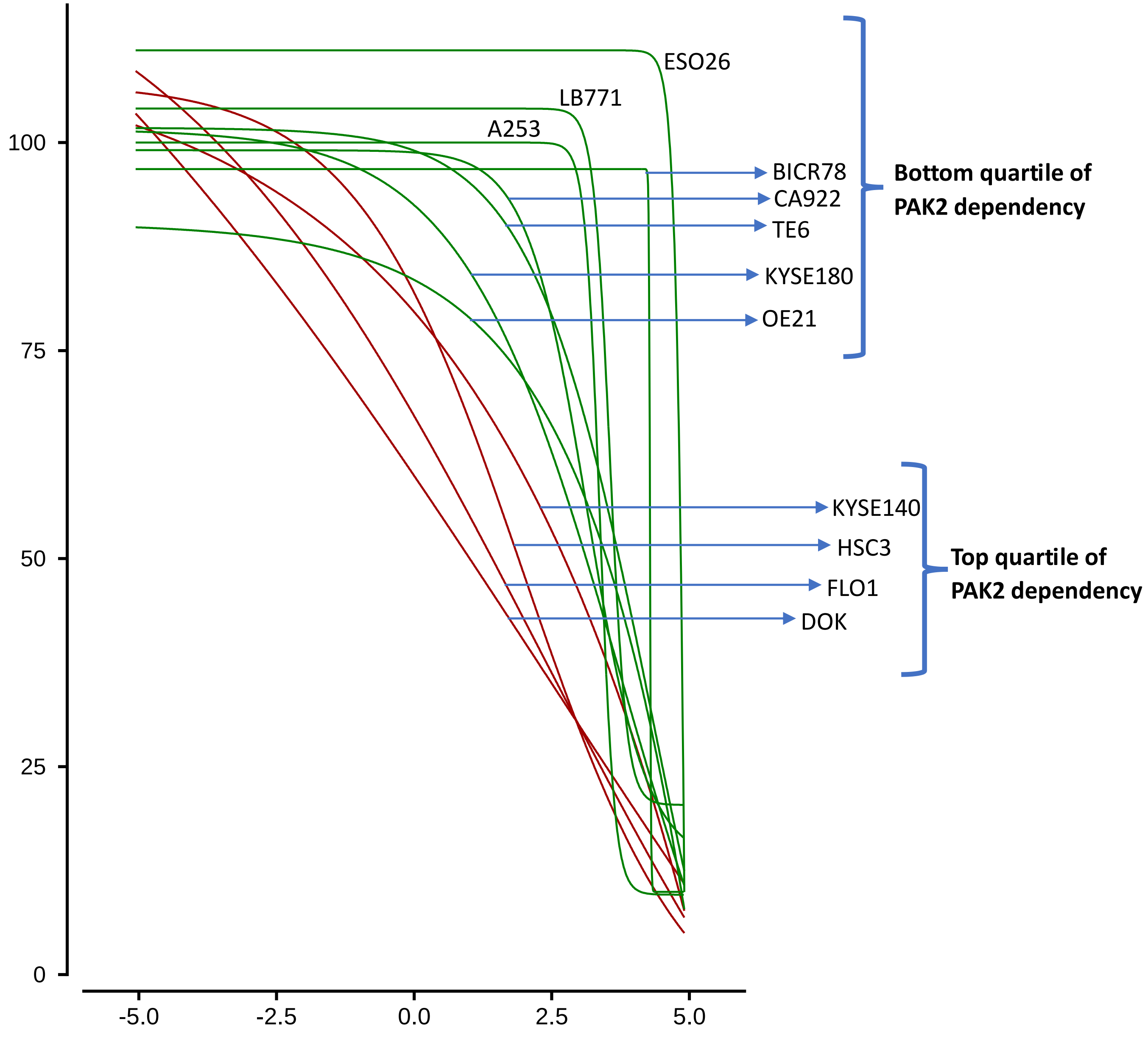

Supplementary Figure 2: **PAK-5339 dose responses of HNSCC models in top vs. bottom quartile of *PAK2* gene effect score**: GDSC-derived data for viability of cell lines in the top vs. bottom quartile of *PAK2* dependency after 72 hours treatment with 7 half-log dilutions of PAK-5339 (0.03 to 30 µM). Cell viability in the screen was determined by Promega CellTiter-Glo™ assay.

Supplementary Figure 3

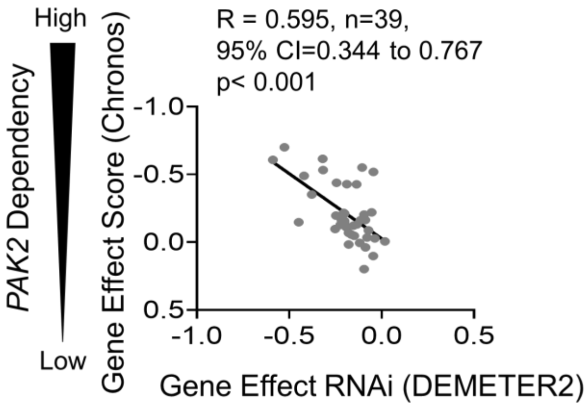

Supplementary Figure 3: **PAK2 dependency in HNSCC models based on CRISPR vs RNAi screening.**

RNAi effect scores were obtained from the DEMETER2 algorithm in DepMap. Pearson correlation coefficients were used to calculate r values, and p value was determined by t distribution.

Supplementary Figure 4:

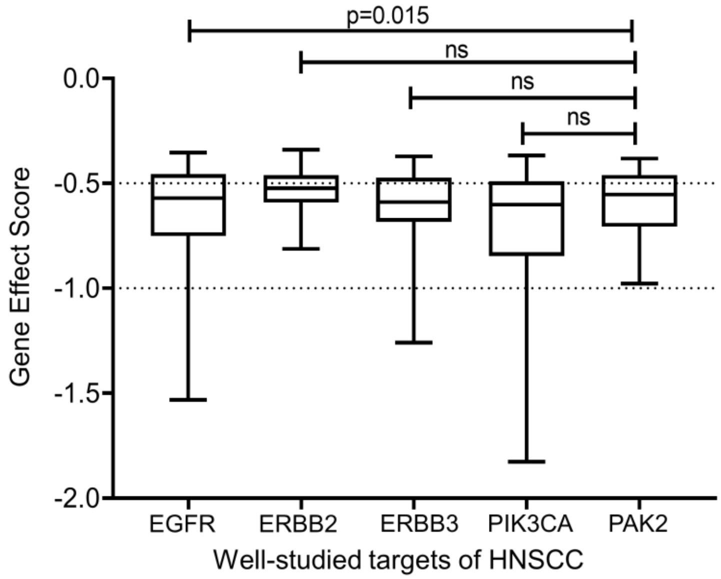

Supplementary Figure 4: Gene effect score distribution for PAK2 vs. well-studied targets in HNSCC. Box plots show distribution of gene effect scores in the HNSCC models meeting the dependency threshold. Adjusted p values were defined by one-way Welch's ANOVA corrected with Dunnett's multiple comparisons test.

Supplementary Figure 5:

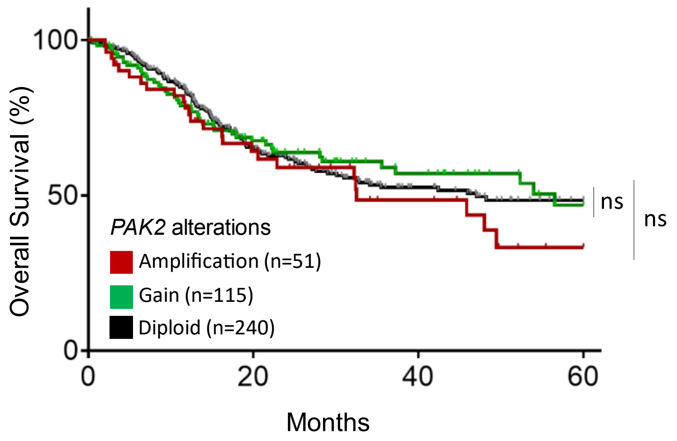

at risk

Amplification	51	39	23	13	8	2
Gain	115	84	48	32	21	12
Diploid	240	184	103	64	45	30
	0	12	24	36	48	60

Supplementary Figure 5: *PAK2* copy number alteration vs. survival of HPV(-) HNSCC patients in TCGA. Kaplan Meier analysis of overall survival in the HPV(-) HNSCC patients with or without copy number alterations in *PAK2*. Survival between groups was compared by log-rank test.

Supplementary Figure 6:

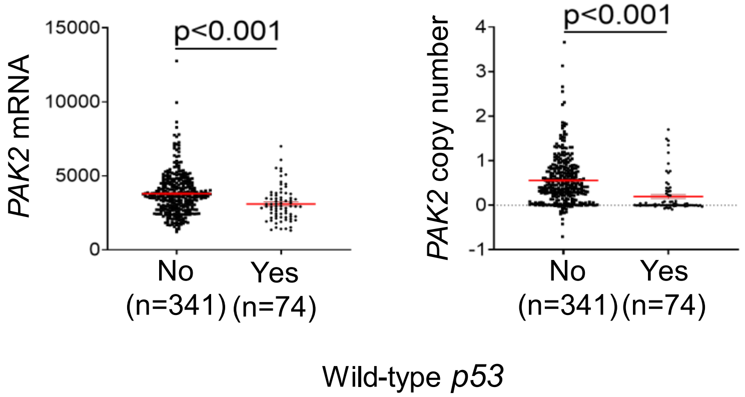

Supplementary Figure 6: *TP53* mutation status vs. PAK2 expression and copy number in HPV(-) HNSCCs in TCGA. The 415 HPV(-) HNSCCs in TCGA segregated by *TP53* mutation status are compared for *PAK2* mRNA and copy gains. P values are calculated using unpaired t test with Welch's correction.

Supplementary Figure 7:

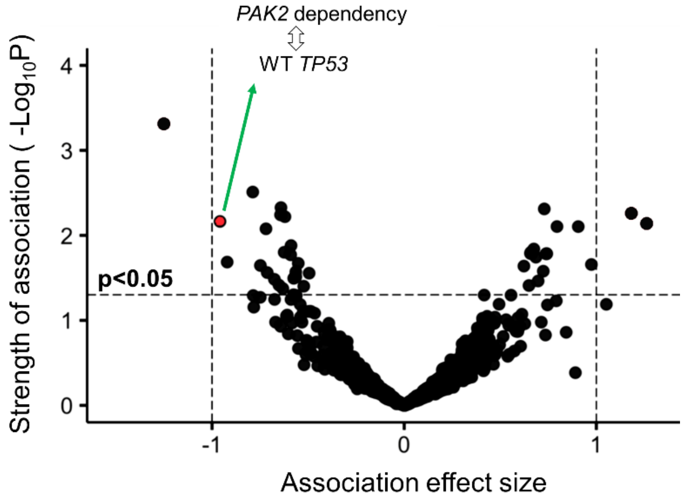

Supplementary Figure 7: Retained association of PAK2 dependency with TP53 WT status in 23Q2 data release. The 143 prioritized targets derived from 23Q2 dependency data were analyzed for associations with common mutations found in the HNSCC models as in Figure 3a.
